# Does Lower Limb Exercise Worsen Renal Artery Hemodynamics in Patients with Abdominal Aortic Aneurysm?

**DOI:** 10.1371/journal.pone.0125121

**Published:** 2015-05-06

**Authors:** Anqiang Sun, Xiaopeng Tian, Nan Zhang, Zaipin Xu, Xiaoyan Deng, Ming Liu, Xiao Liu

**Affiliations:** 1 Key Laboratory for Biomechanics and Mechanobiology of Ministry of Education, School of Biological Science and Medical Engineering, Beihang University, Beijing, China; 2 Radiologic Department, Beijing Anzhen Hospital, Capital Medical University, Beijing, China; 3 College of Animal Science, Guizhou University, Guiyang, China; Medical University Innsbruck, AUSTRIA

## Abstract

Renal artery stenosis (RAS) and renal complications emerge in some patients after endovascular aneurysm repair (EVAR) to treat abdominal aorta aneurysm (AAA). The mechanisms for the causes of these problems are not clear. We hypothesized that for EVAR patients, lower limb exercise could negatively influence the physiology of the renal artery and the renal function, by decreasing the blood flow velocity and changing the hemodynamics in the renal arteries. To evaluate this hypothesis, pre- and post-operative models of the abdominal aorta were reconstructed based on CT images. The hemodynamic environment was numerically simulated under rest and lower limb exercise conditions. The results revealed that in the renal arteries, lower limb exercise decreased the wall shear stress (WSS), increased the oscillatory shear index (OSI) and increased the relative residence time (RRT). EVAR further enhanced these effects. Because these parameters are related to artery stenosis and atherosclerosis, this preliminary study concluded that lower limb exercise may increase the potential risk of inducing renal artery stenosis and renal complications for AAA patients. This finding could help elucidate the mechanism of renal artery stenosis and renal complications after EVAR and warn us to reconsider the management and nursing care of AAA patients.

## Introduction

An abdominal aortic aneurysm (AAA) is a localized, balloon-like dilatation of the abdominal aorta. A progressive enlargement of the aneurysm may lead to rupture and internal bleeding. Approximately 5–7% of the older population, primarily males over the age of 60, are at risk of developing AAAs [1]. Ruptured AAAs are the 13^th^ leading cause of death in the United States, with a mortality rate of up to 75% [2].

Endovascular aneurysm repair (EVAR) is a less invasive alternative to open surgical repair and is a mechanical solution to the issues of progressive expansion and rupture of AAAs [3,4]. Trials have demonstrated that EVAR has lower mortality rates than open surgical repair, especially in the recovery stage post-operation [5]. However, with the widespread use of EVAR, additional problems have emerged, such as renal artery stenosis (RAS) and renal dysfunction [5–8]. In a previous study of 6516 AAA patients undergoing EVAR, post-procedure acute renal failure developed in 439 cases (6.7%) [9].

Studies have suggested that artery stenosis and renal dysfunction have close relationships with the local hemodynamic characteristics of arterial and renal perfusions [6,10,11,12]. Disturbed and non-physiological blood flows may interfere with endothelial metabolic functions and retard the transport of atherogenic substances, such as low density lipoproteins (LDLs), away from the wall. Subsequently, this enhances the intimal accumulation of lipids and hence, increases the vulnerability of these regions to atherosclerosis. Renal hypoperfusion, which is characterized with a decrease in blood flow rate, initiates a process of intra-renal structural and functional changes with a progressive loss of renal mass. Ischemic nephropathy may occur as a result of a partially compromised vascular supply.

To analyze the induction of potential renal artery stenosis and renal dysfunction due to the altered hemodynamics in the renal artery from EVAR, Sun and his colleagues reported the effect of stents in the renals in FEVAR and the effect of the stent-material in suprarenal stent-grafts [13,14]. Based on the assumption that the position of the stent-graft would inevitably change the geometry of the renal ostia and disturb blood flow in the renal arteries, they found that the effect of the stent-graft and the fenestrated stent wires on the renal blood flow was minimal. Additionally, no significant change in the wall shear stress (WSS) was noticed post-model. Therefore, the mechanisms of renal stenosis and kidney dysfunction after EVAR remain unknown and require further investigation.

When a patient performs lower limb exercises, more blood flows into the abdominal aorta. The enhanced blood perfusion in the abdominal aorta stabilizes blood flow in AAA and enhances the WSS on the inner wall of the abdominal aorta and is conducive to preventing the development of AAA [15–17]. However, as blood perfusion increases to the abdominal aorta, the subsequent branched flow to the renal arteries decreases accordingly, even to approximately 70% of flow during rest [18]. Based on these observations of previous studies, the mechanisms of the hemodynamic response in renal arteries to lower limb exercises remain unknown. Correspondingly, the potential influence on the physiological functions of renal artery and renal system has yet to be determined.

The authors of the present study hypothesized that the participation in lower limb exercises decreases the blood flow velocity and changes the hemodynamic environment in the renal arteries and thereby, negatively influences the renal artery physiology and the renal function of EVAR patients.

To test this hypothesis, pre- and post-operative EVAR models of AAAs were reconstructed based on CT scan images. The hemodynamic environment in an AAA and the renal arteries were numerically simulated under conditions of rest and lower limbs exercise. The main purpose of this study was to investigate the influence of low limb exercise on the hemodynamics of renal arteries. The implications of this study would help in the determination of potential causes of renal artery complications after EVAR.

## Materials and Methods

### Artery model

**Fig 1:**
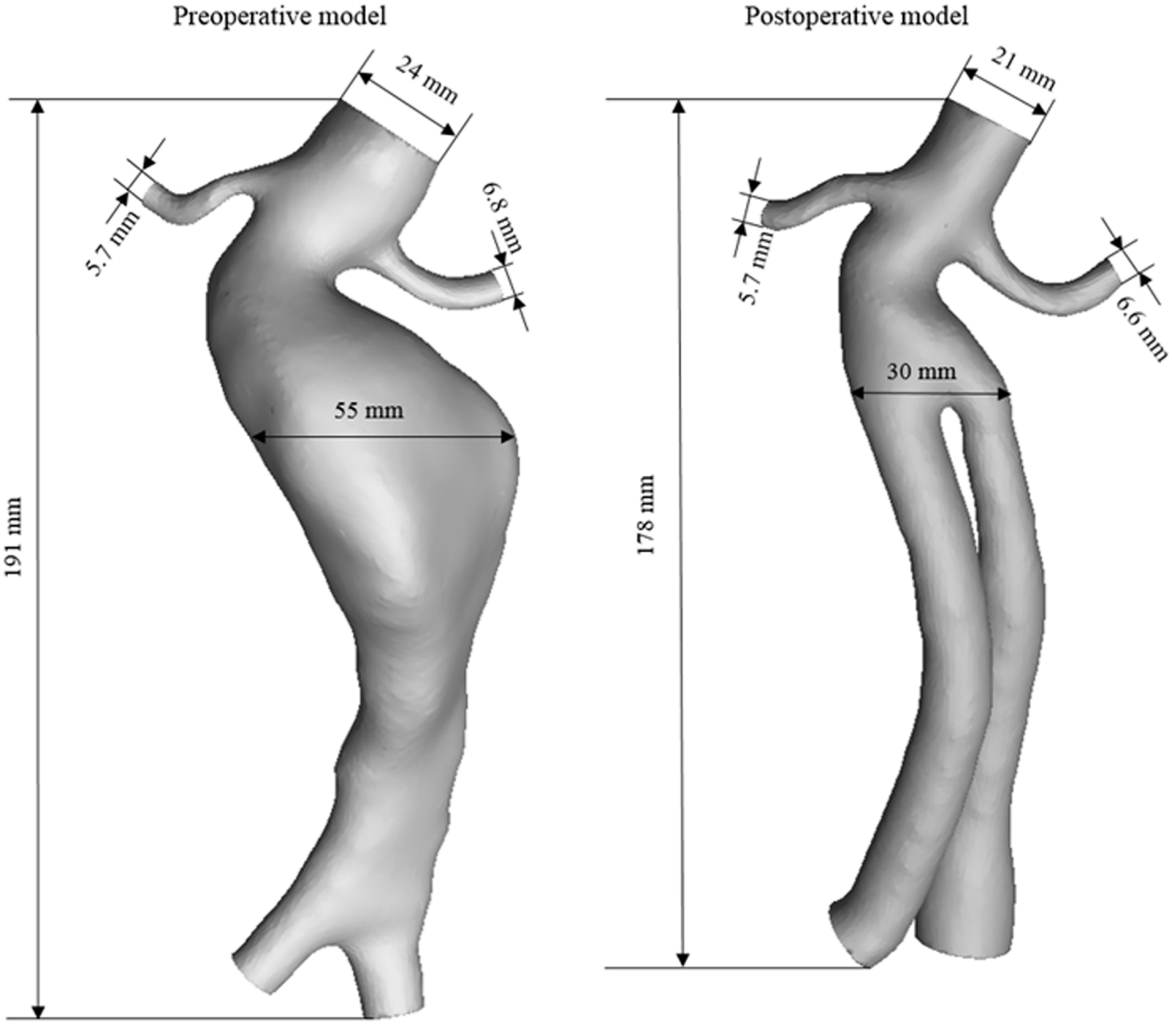
Pre- and post-operative artery models reconstructed from CT data. The abdominal artery was defined as inlet, whereas bilateral renal arteries and iliac arteries were set as outlets.

To simulate the realistic hemodynamic conditions of the abdominal aorta and the renal artery, arterial models were reconstructed based on the computed tomography (CT) scan images obtained from Beijing Anzhen Hospital (Beijing, China). The CT-relevant parameters were as follows: 0.5 mm slice thickness, 1.5 mm reconstruction spacing/increment, 0.5 mm slice overlap and a 512×512 image resolution. These images were used to reconstruct models with Mimics software (v9.0, Materialise, Ann Arbor, MI, USA). Simple smooth processing was applied to the models and Rapidform (v2004, INUS, Korea) and Solidworks (Solidworks Corporation, Boston, MA, USA) were used for further processing. The pre- and post-operative models are shown in Fig 1.

### Mesh generation

**Fig 2:**
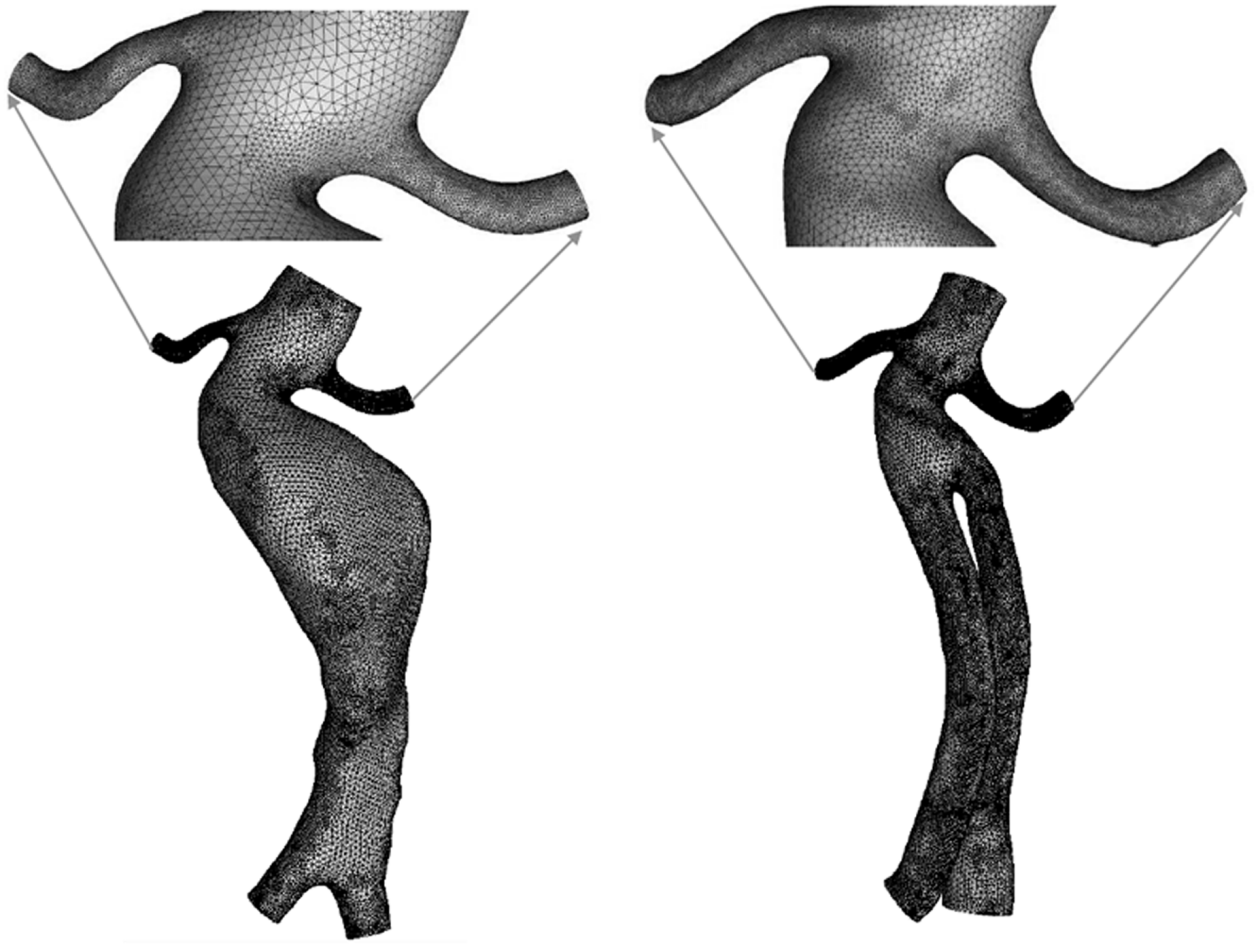
Meshes of the pre- and post-operative models. Both models were meshed with the exact same parameters: mixed tetrahedral and hexahedral volume meshes, 0.2 mm minimum size and 1.0 mm maximum size, 5 boundary layer and 0.2 mm total height.

Meshes were generated using ICEM (ANSYS, Inc., Canonsburg, PA, USA). The surfaces of the models were meshed using a mixture of tetrahedral and hexahedral volume meshes (Fig 2). The maximum and minimum sizes of the mesh were 1.0 mm and 0.2 mm, respectively, for the pre- and post-operative models. The node numbers were 1,549,630 and 1,037,579 for the pre- and post-operative models, respectively. The number of the boundary layer was set to 5, the height ratio was set to 1.3, and the total height was set to 0.2 mm.

### Boundary conditions

**Fig 3:**
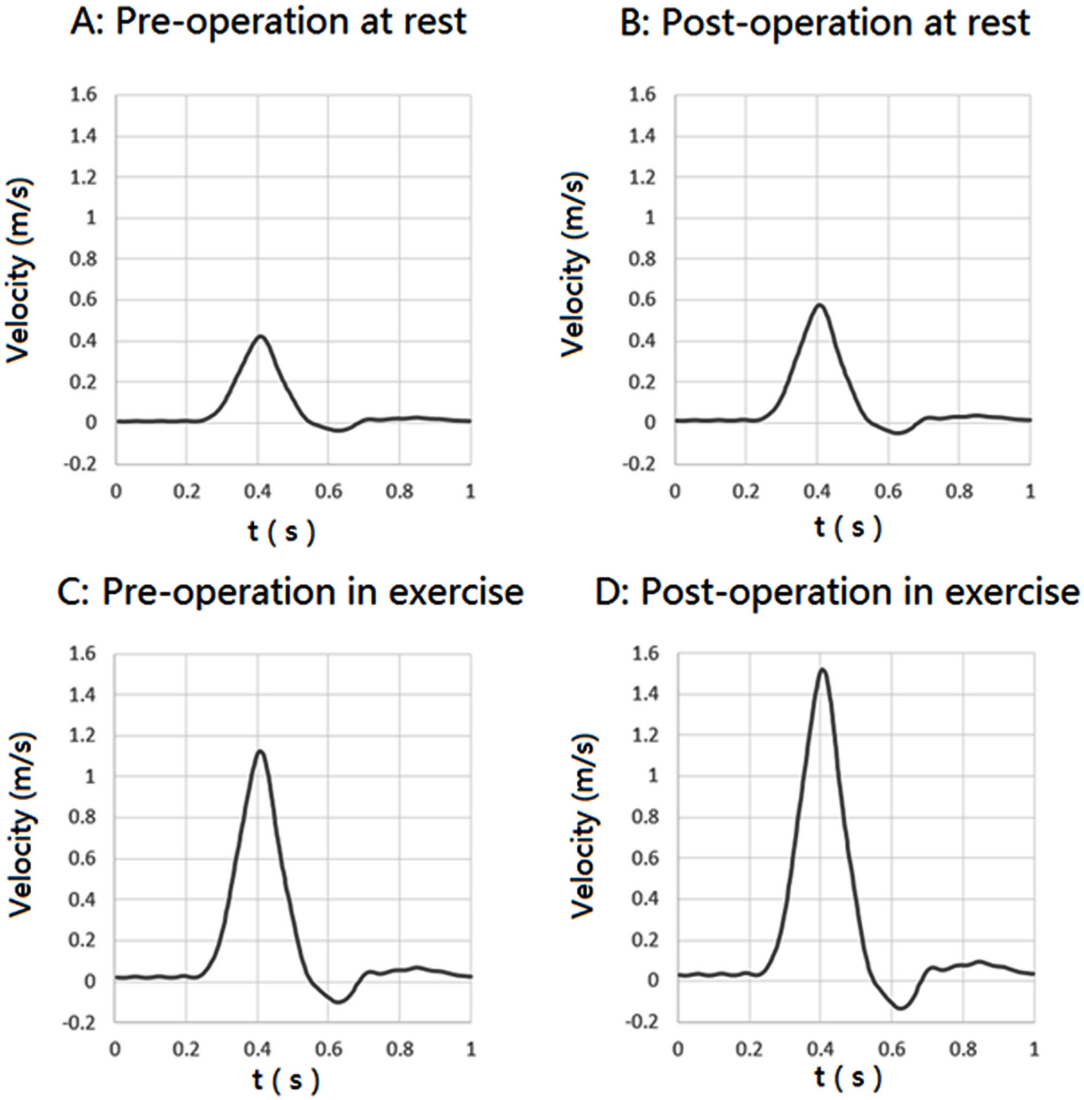
Inlet velocity pulse.

Simulations were computed under pulsatile flow conditions, and the inlet flow wave was calculated according to the studies conducted by Walsh PW and Li Z [2, 18]. Fig 3 shows four inlet pulsatile flow waves that were used for the pre-operative model at rest, the pre-operative model with exercise, the post-operative model at rest and the post-operative model with exercise. Table 1 shows the mean flow velocities at the inlet for these four conditions.

**Table 1:** Mean velocities at the inlet used in this study.

Models	Mean velocities (m/s)	
	Rest	Exercise
Pre- operative	0.0648	0.171
Post- operative	0.0879	0.232

The *outflow* condition represents a fully developed flow and was used for all outlets. Because lower limb exercise leads to significant alterations of the flow ratios through the four outlets, different flow ratios were used for the rest and exercise conditions according to Walsh PW’s previous study [18]. For the rest condition, a constant flow ratio of 26% was applied at both renal outlets and 24% was applied at both iliac outlets. For the exercise condition, a constant flow ratio control of 7.1% was applied at both renal outlets and 42.9% was applied at both iliac outlets.

### Assumptions and governing equations

Blood was modeled as a Newtonian fluid and assumed to be homogeneous and incompressible [19,20]. The numerical simulation was based on a three-dimensional incompressible Navier-Stokes equation and the conservation of mass:

$$\rho \left[\frac{\partial \overset{⃑}{u}}{\partial t}+\left(u∙\nabla \right)\overset{⃑}{u}\right]+\nabla p-\mu \nabla^{2}\overset{⃑}{u}\;=\;0$$ (1)

$$\nabla ∙\overset{⃑}{u}\;=\;0$$ (2)

where $\overset{⃑}{u}$ and *p* respectively represent the fluid velocity vector and the pressure. *ρ* and *μ* are the density and viscosity of blood (*μ* = 3.5 × 10^-3^ *kg*/*m s* and *ρ* = 1050*kg*/*m* ^3^). The Finite Volume Method of the CFD software package, ANSYS Fluent 14.0 (ANSYS, Lebanon, NH, USA) was used for the simulations. The SIMPLEC algorithm was applied for the coupling of the outflow velocity terms, and a segregated algorithm was applied to solve all equations. The convergence criterion was set to 1×10^−5^. Six cycles were required to obtain a convergence for the transient analysis, with 200 steps in each cycles (T = 1 s). The whole computational process spanned two weeks.

### WSS-based hemodynamic quantities

Post-processing was conducted using a MATLAB programming environment (The MathWorks, Natick, Mass). Three WSS-based hemodynamic parameters (TAWSS, OSI and RRT) were calculated based on a study by Claudio Chiastra [21].

The time-averaged wall shear stress (TAWSS), was used to describe the features of WSS in pulsatile flow. The TAWSS was defined as follows:

$$\;TAWSS\;=\;\frac{1}{T}\int_{0}^{T}\left|WSS(s,t)\right|∙dt,$$ (3)

where *T* is the duration of the cardiac cycle and *s* is the position on the vessel wall.

The oscillatory shear stress index (OSI) on the inner wall of the models was calculated as:

$$OSI\;=\;0.5\left[1-\left(\frac{\left|\frac{1}{T}\int_{0}^{T}WSS(s,t)∙dt\right|}{\frac{1}{T}\int_{0}^{T}\left|WSS(s,t)\right|∙dt}\right),\right]$$ (4)

where WSS is a vector parameter and its direction changes with the cardiac cycle time. The OSI denotes the changing frequency of the WSS direction, which ranges from 0, where the flow is one-directional without oscillations, to 0.5, where the WSS direction frequently changes.

The relative residence time (RRT) was calculated as:

$$RRT\;=\;\frac{1}{(1-2∙OSI)∙TAWSS}$$ (5)

RRT was used to determine the residence time of particles near the wall and recommended as a single metric of low and oscillating shear stress. It is inversely proportional to the magnitude of the TAWSS vector and has obvious connections to the biological mechanisms of atherosclerosis.

## Results

Because the study focuses on the effect of lower limb exercise on the hemodynamics of renal arteries, the detailed results included parameters related to the flow velocity, WSS, TAWSS, OSI and RRT, especially in renal arteries.

### Flow velocity

**Fig 4:**
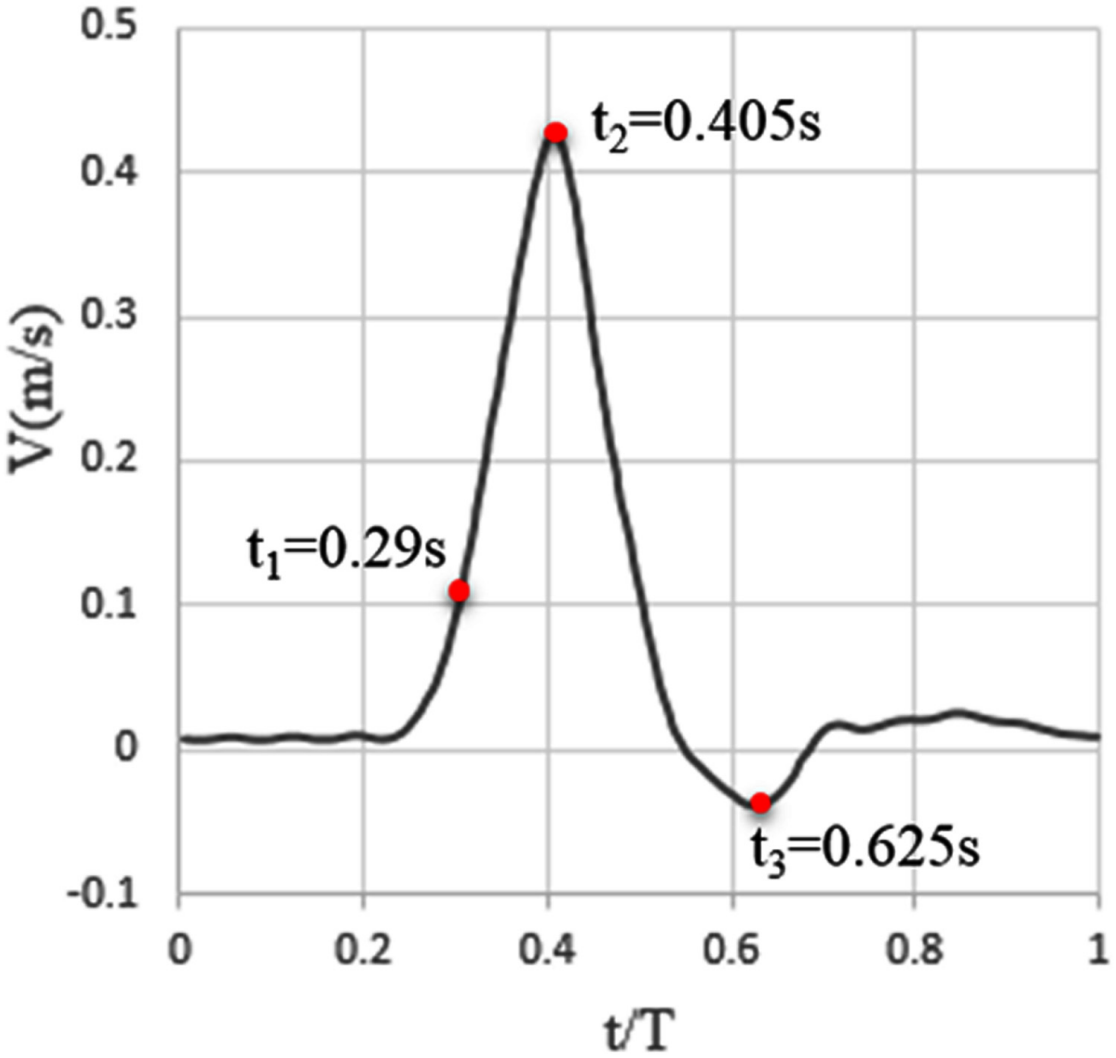
Three typical points during one cardiac cycle. t_1_, t_2_ and t_3_, respectively represent early systole, peak systole and end systole (only one flow pulse wave is shown).

Three points from one cardiac cycle, early systole (t_1_), peak systole (t_2_) and end systole (t_3_), were selected and are shown in Fig 4. The hemodynamic results at these three typical phases will be presented.

**Fig 5:**
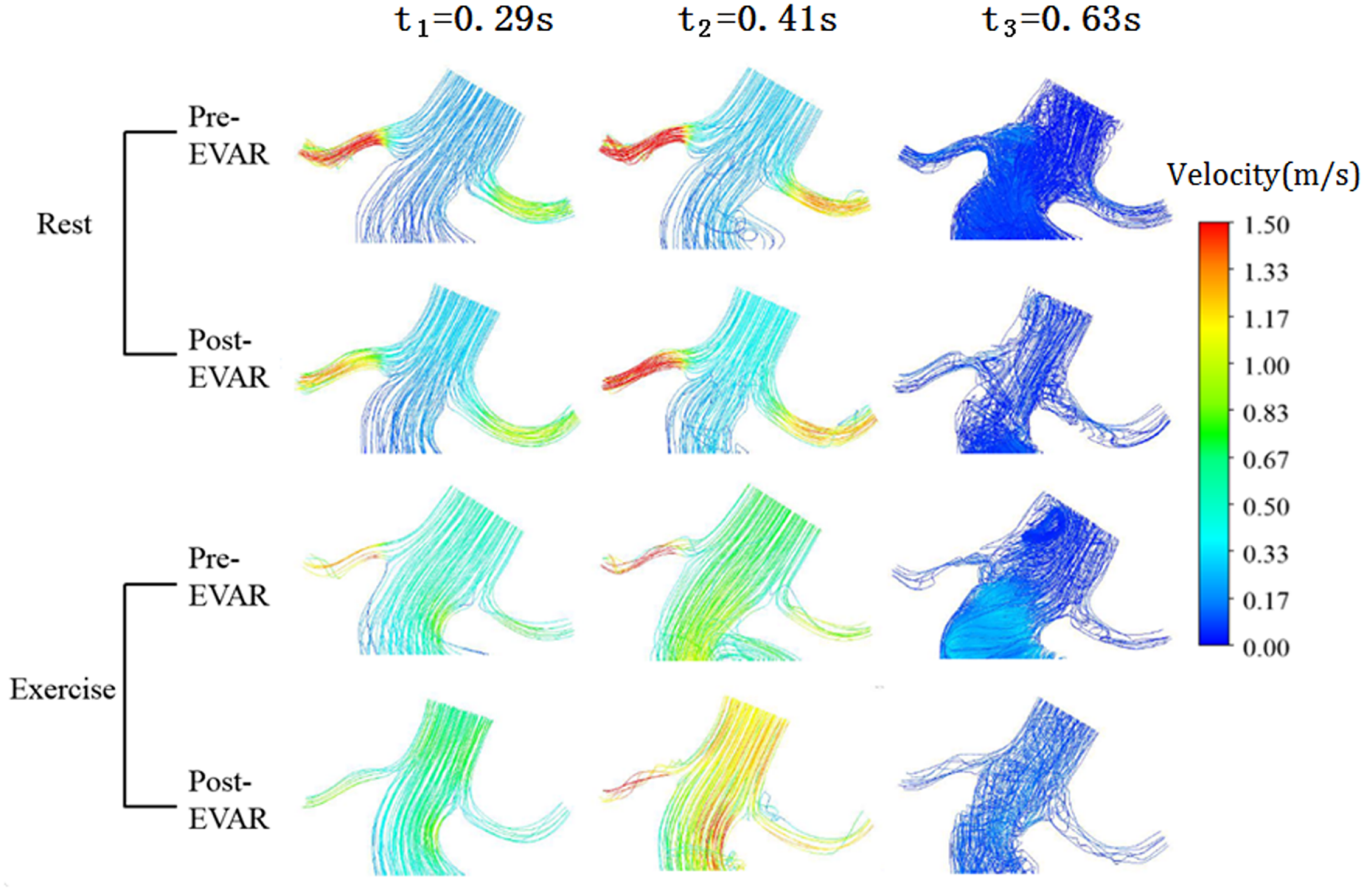
The streamlines near renal arteries at three typical points.

Fig 5 shows the velocity streamlines near the renal ostium, with the magnitude of the flow velocity indicated in color. There was no obvious difference in the flow patterns between models. Disturbed flow appeared at time t_3_ (end systole). However, under rest conditions, the flow velocity in the renal arteries was higher than that under exercise conditions. Exercise had a significant influence on the velocity in renal arteries.

In this patient-specific model, the distorted artery geometry caused different hemodynamic features in the bilateral renal arteries. The flow velocity in the right renal artery was greater than that in the left renal artery.

Multiple vortices were observed at time t_3_ under both rest and exercise conditions. Regions of low flow velocities and recirculation zones that existed throughout the whole model under rest conditions were replaced by higher flow velocities and more complex recirculation zones under exercise condition.

### Wall shear stress (WSS) and time-averaged WSS (TAWSS)

**Fig 6:**
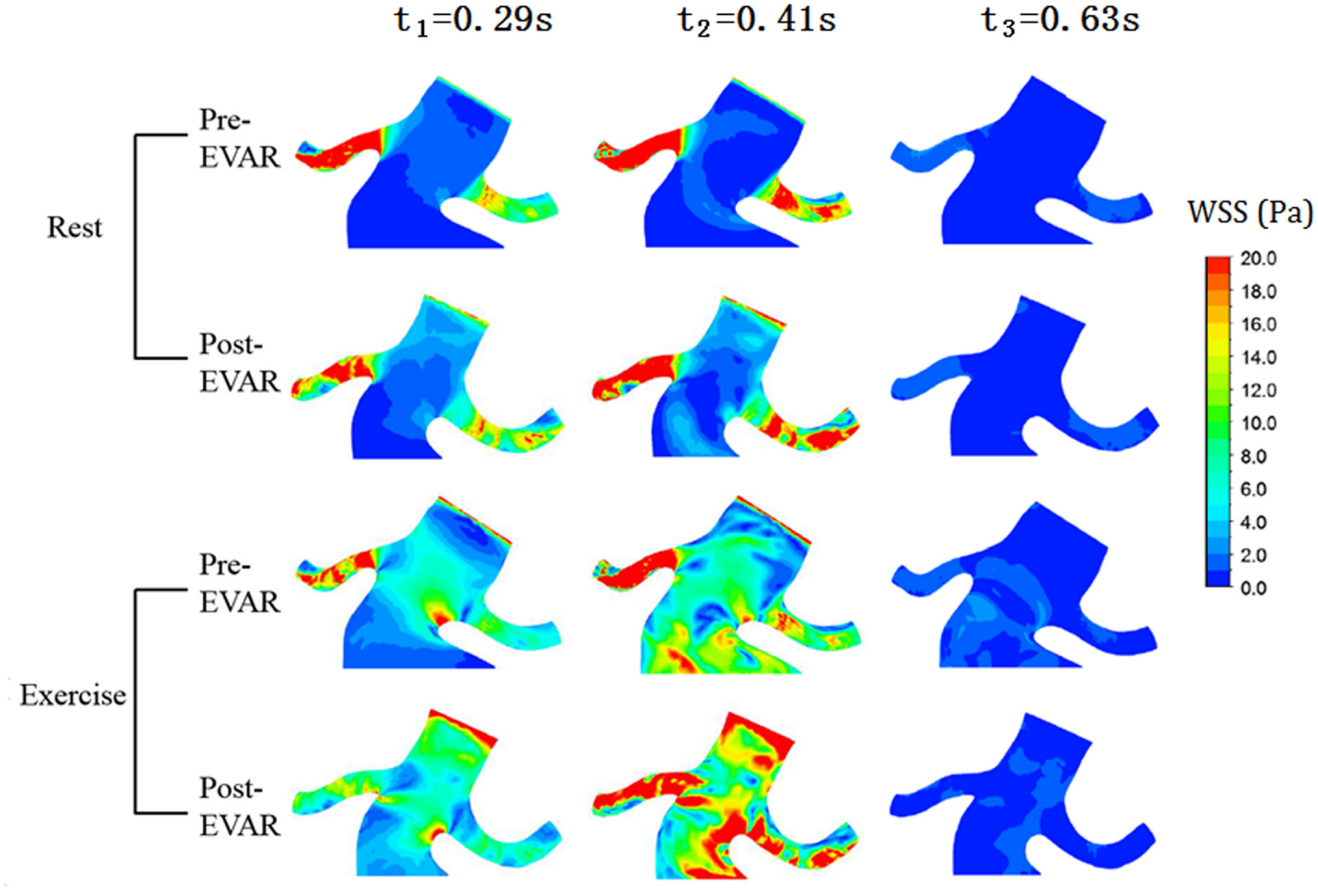
Contours of WSS. The WSS on the renal artery wall was significantly decreased, especially at t_1_ and t_2_.

**Fig 7:**
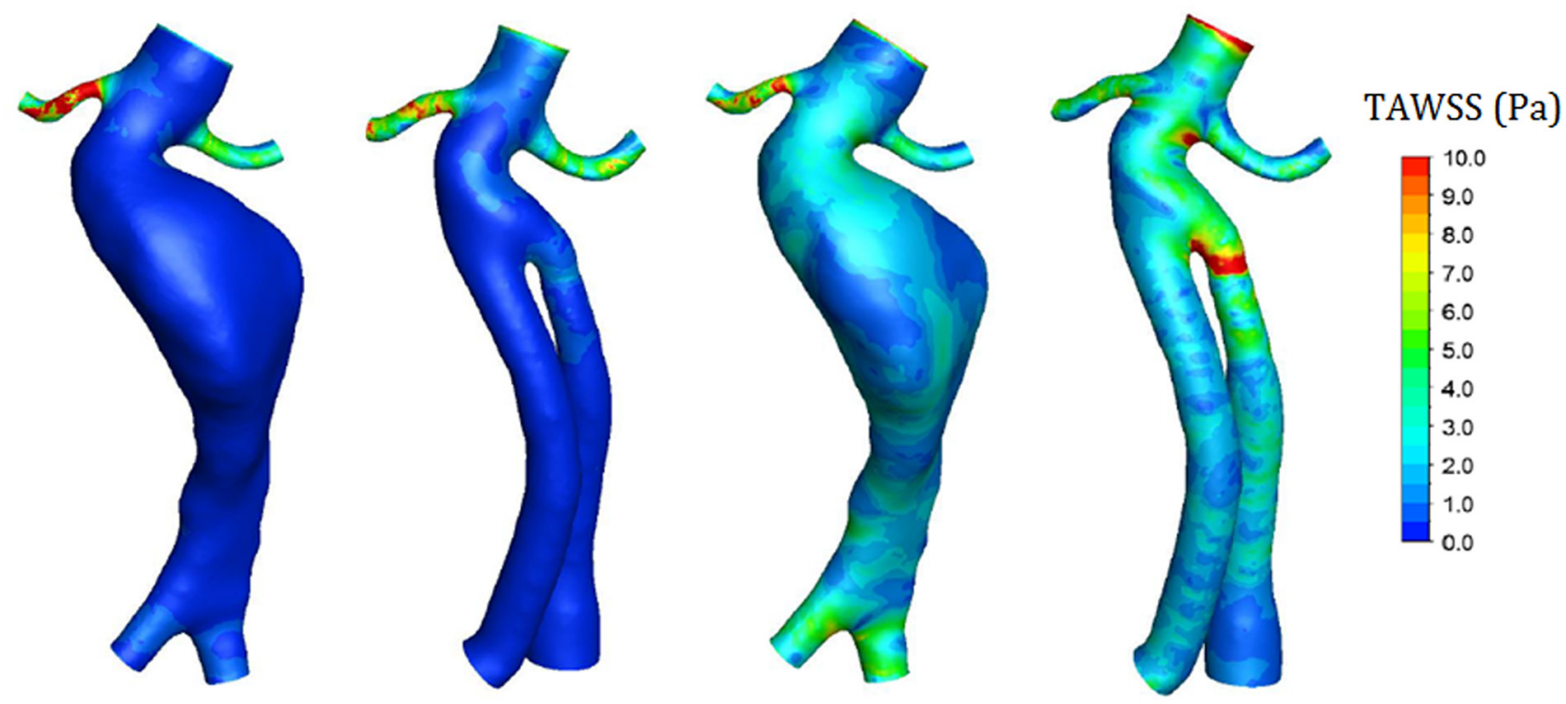
Contours of TAWSS. Exercise increased the TAWSS on the aneurysm and stent-graft and caused notable TAWSS reduction on the right renal artery. (a) Pre-operative in rest, (b) post-operative in rest, (c) pre-operative in exercise, (d) post-operative in exercise.

Contours of the WSS and the TAWSS at the three points are shown in Figs 6 and 7. Exercise decreased the WSS on the renal arterial wall and increased the WSS on aneurysms and stent-grafts. Under rest conditions, the average WSS on the right renal artery of a post-operative model was 13.9 Pa at time t_1_ and 20.9 Pa at time t_2_. Under exercise conditions, the average WSS was reduced to 8 Pa at time t_1_ and 16 Pa at time t_2_. However, under rest conditions, the average WSS on the stent-graft of the post-operative model was 1.3 Pa at time t_1_ and 0.7 Pa at time t_2_. Under exercise conditions, it increased to 5.9 Pa at time t_1_ and 10.7 Pa at time t_2_.

Similar observations were made for the TAWSS distribution as for the above-described WSS distribution. The TAWSS on the renal artery was relatively higher under rest conditions than in exercise conditions. The TAWSS significantly increased on the aneurysm wall and the graft wall under lower limb exercises. The TAWSS on left renal artery was relatively constant for the different conditions.

### Oscillatory shear index (OSI) and relative residence time (RRT)

Fig 7 shows the contours of the OSI under different conditions. Generally, areas of high OSI were observed in areas with low WSS. In the TAWSS distribution (Fig 6), the high WSS areas, such as the stent joint region and the renal artery, especially right renal artery, exhibited low OSI. The low TAWSS areas, such as the stent-graft and aneurysm site, especially at points along the aneurysm with large diameters, exhibited high OSI.

**Fig 8:**
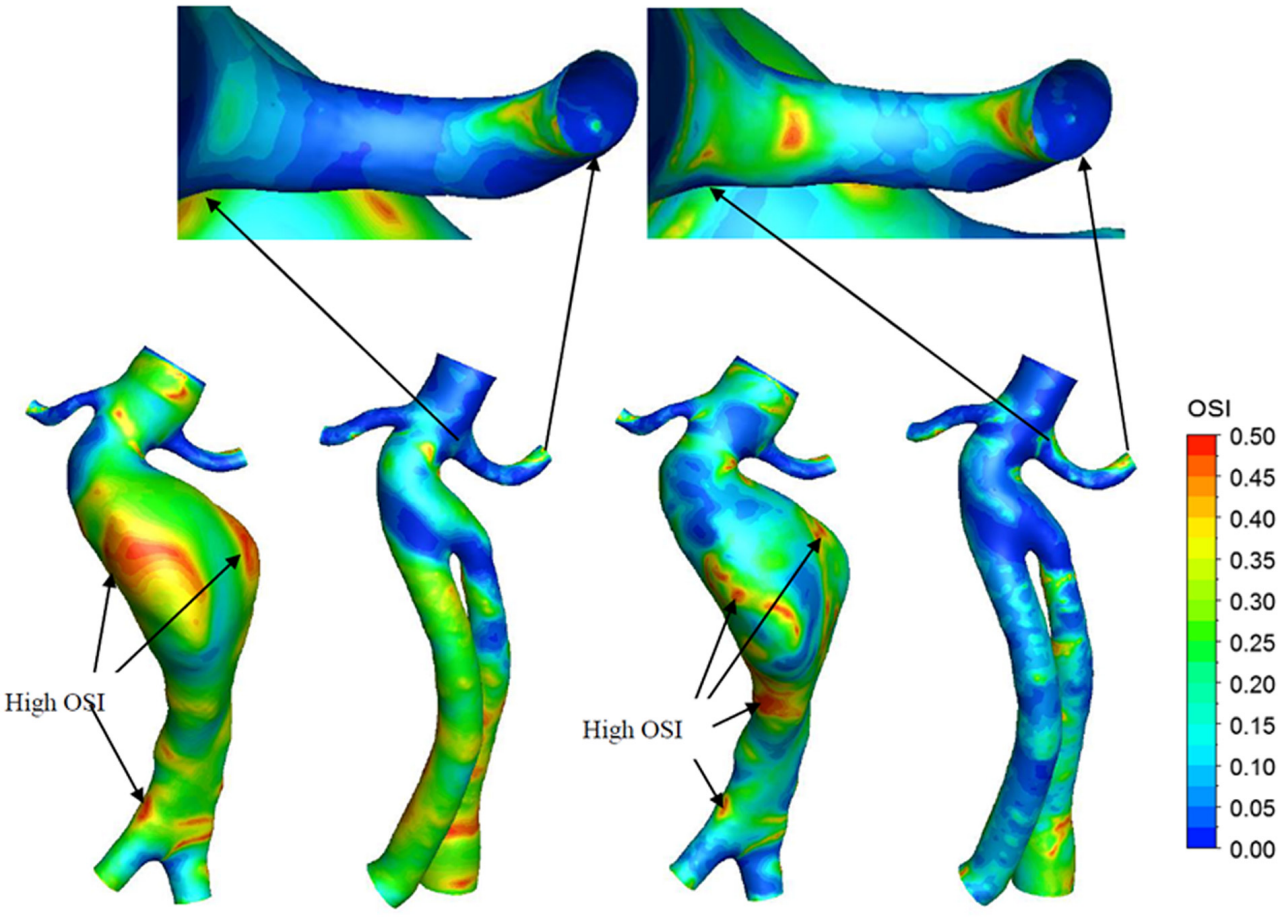
Contours of OSI. The left renal artery of Post- operative model were partially enlarged. (a) Pre-operative in rest, (b) post-operative in rest, (c) pre-operative in exercise, (d) post-operative in exercise.

Exercise increased the OSI in the upper surface of the renal artery, especially in the left renal artery. As observed from the enlarged image in Fig 8, the OSI in the left renal artery increased under exercise conditions. However, exercise conditions reduced the OSI at the aneurysm site and at the wall of the stent-graft in the pre- and post-operation models.

**Fig 9:**
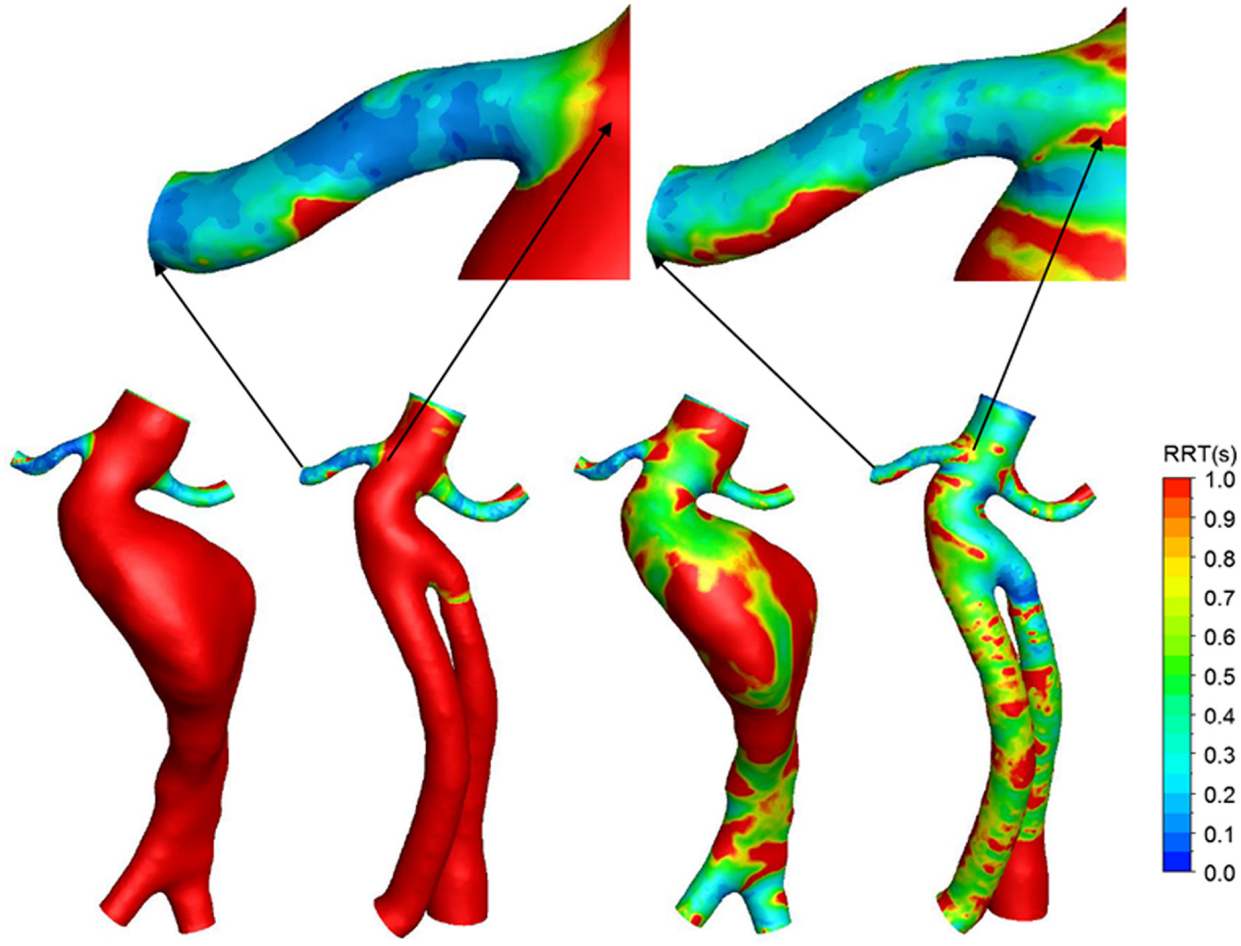
Contours of relative residence time (RRT). The right renal artery of Pre-operative and Post-operative model were partially enlarged. (a) Pre-operative in rest, (b) post-operative in rest, (c) pre-operative in exercise, (d) post-operative in exercise.

The contours of RRT under the four conditions are shown in Fig 9. The exercise condition showed increased RRT values in the renal arteries of the pre-operative and post-operative models. The RRT in the aorta and the stent-graft significantly decreased. For example, exercise induced an apparent rise of the RRT in the right renal artery. The enlarged image shows an apparent increase in the RRT in the right renal artery of the post-operative model (Fig 9).

## Discussion

Post-operative renal artery stenosis and renal dysfunction have attracted more and more attentions.[22] The causes for these complications remain unknown, and studies have been conducted to investigate the relationship between EVAR and the resultant complications. A few studies have focused on the altered hemodynamic environment in the renal artery caused by stent-graft intervention. A few possible factors have been proposed, such as a fenestrated vessel stent placement [13] and the presence of suprarenal stent wires [14]. However, these studies demonstrated that EVAR had a less significant hemodynamic effect on the renal arteries than predicted.

In this study, we investigated lower limb exercise as another potential factor for renal complications after EVAR. This investigation was inspired by previous studies that were evaluating hemodynamic changes in AAA [15,23]. We reconstructed pre-operative and post-operative models of AAA under rest and lower limb exercise conditions. Between these two conditions, there was an apparent difference in the division of blood flow to the renal arteries and the abdominal aorta. A higher blood perfusion rate to the iliac arteries through the abdominal aorta was observed under the lower limb exercise condition. This indicated that the flow rate in the renal arteries significantly decreased under the exercise conditions. The flow rate change directly induced a decrease in the WSS in the two renal arteries. Meanwhile, the OSI increased in the renal arteries, which indicated a more oscillatory flow profile. The RRT also increased in the renal arteries, which increased the contact and adherence times for lipids or emboli to interact with the renal artery wall.

Because low WSSs, high OSIs and high RRTs are all indicators of arterial stenosis and atherosclerosis [24], lower limb exercises would favor a hemodynamic environment in the renal arteries that promoted these conditions. Because the onset of AAA traditionally has strong clinical associations with aging, smoking, and atherosclerosis, [25–27], AAA patients have increased risks for atherosclerotic events. Therefore, the authors speculated that lower limb exercise would increase the risk of renal artery stenosis and atherosclerosis in AAA patients. To valid this speculation, more experiments and clinical evaluations should be conducted in the future.

When the results of the pre-operative and post-operative models were compared, we found that EVAR reduced the WSS in the renal arteries under rest and exercise conditions. EVAR also slightly increased the OSI and the RRT in the post-operative model under rest and exercise conditions. The stent-graft intervention used in EVAR seemed to further deteriorate the hemodynamic environment of the renal artery.

Ga-Young Suh suggested that mild lower limb exercise may be sufficient to reduce the oscillatory and stagnant hemodynamic conditions in AAA [23]. However, the present study illustrated that lower limb exercise could worsen the renal artery hemodynamic environment, especially after EVAR, and increase the risk of renal artery stenosis. This study could help elucidate the mechanism of renal artery complications after EVAR and additionally, reevaluate the recovery care methods for AAA patients.

The morphology of an AAA varies from patient to patient. Thus, the hemodynamic environment also varies. The aorta artery model used in this study has a prominent curve at the neck of the aneurysm, and the two renal arteries are asymmetrical. The asymmetric geometry induced very different hemodynamic characteristics in the two renal arteries. This phenomenon could be directly embodied in the results: the velocity and the WSS of the right renal artery was greater than those in the left renal artery, and the RRT of the right renal artery was lower than that in the left renal artery. More patient-specific model reconstructions are necessary to determine a generalized model. However, for the purpose of clinical use, individualized analyses would be essential.

## Conclusion

This study investigated the relationship between lower limb exercise and the renal hemodynamic environment. The study concluded that lower limb exercise worsens the hemodynamic environment in the renal artery and has potential negative influences on the renal artery and kidney physiologies, especially after EVAR. This study could help elucidate the mechanism of renal artery stenosis and renal complications after EVAR, and it also serves as warnings of possible problems for the AAA patients. Some of the present nursing care strategies for AAA patients, which encourage patients to have exercises, are suggested to be reconsidered and evaluated.
